# Strengthening healthcare system resilience: a comprehensive framework for tropical cyclone preparedness and response

**DOI:** 10.1016/j.lana.2025.101205

**Published:** 2025-07-28

**Authors:** Attila J. Hertelendy, Caleb Dresser, Sophia Gorgens, Arabella P. Hertelendy, Paul D. Biddinger, Gregory Ciottone

**Affiliations:** a Department of Information Systems and Business Analytics, College of Business, Florida International University, Miami, FL, USA; b Disaster Medicine Fellowship, Department of Emergency Medicine, Beth Israel Deaconess Medical Center, Boston, MA, USA; c College of Public Health and Health Science, University of Florida, Gainesville, FL, USA; d Center for Disaster Medicine, Mass General Brigham, Boston, MA, USA; e Harvard Medical School, Boston, MA, USA; f Department of Environmental Health, Harvard T.H. Chan School of Public Health, Boston, MA, USA

**Keywords:** Health systems resilience, Tropical cyclone, Hurricane preparedness, Framework, Hospital disaster preparedness, Flooding

## Abstract

This review examines healthcare system resilience to tropical cyclones through complementary frameworks of temporal phases (Before-During-After) and geographic contexts (Inside-Outside Impact Zone). The paper highlights how climate change is intensifying cyclone threats while demographic transitions create increasingly vulnerable patient populations dependent on continuous healthcare. Despite decreasing immediate mortality from cyclones, research reveals concerning increases in delayed morbidity and mortality due to disrupted healthcare access. Seven critical dimensions of healthcare resilience are identified: maintaining continuity of care for vulnerable populations, transitioning from reactive response to proactive resilience, strategic resource prioritization, adapting to climate change, integrating efforts across phases and zones, ensuring health equity, and addressing research gaps. A tiered approach to strengthening resilience is proposed, from immediate low-resource actions to long-term structural investments. The review emphasizes that healthcare systems must transform from reactive disaster response to proactive resilience strategies to protect vulnerable populations in an increasingly turbulent future climate.

## Introduction

**Fig. 1:**
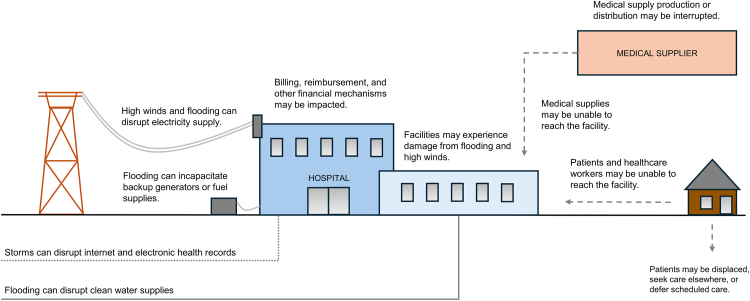
Impacts of tropical cyclones on healthcare system functioning.

Tropical cyclones threaten the functioning of healthcare systems and the well-being of populations that depend on them. Evacuation of hospitals and long-term care facilities, damage to healthcare facilities, and interruptions in access to care create substantial risk of harm to patients (Fig. 1).^1^, ^2^, ^3^, ^4^

Immediate mortality attributed to tropical cyclones declined during the 20th century, thanks to improvements in weather forecasting, tropical cyclone early warning, and evacuation of populations out of harm's way into safer locations ranging from inland cities to elevated cyclone shelters.^5^ However, recent research has shown that subsequent morbidity and mortality in settings that experience tropical cyclones can extend months or years after the storm.^5^, ^6^, ^7^, ^8^ There is evidence that excess deaths following tropical cyclones are now increasing in some regions, including a 21% increase in excess deaths per decade since 1980 in Latin America and the Caribbean.^9^^,^^10^

Emerging threats and improvements in epidemiological understanding call for new approaches from the standpoint of healthcare systems. Climate change, aging and medically complex populations, practical limits to response capacity and financing for healthcare adaptation, and emerging understanding of the prolonged indirect health impacts of these storms create the need for a broader assessment of the role of healthcare systems before, during, and after tropical cyclones.^11^

This article examines healthcare system resilience through two complementary frameworks: temporal phases (Before-During-After) and geographic contexts (Inside-Outside Impact Zone). These frameworks organize our understanding of how healthcare systems can prepare for, respond to, and recover from tropical cyclones while recognizing that impacts extend beyond directly affected areas. Seven key challenges emerge: maintaining continuity of care for vulnerable populations; transitioning from reactive response to proactive resilience; strategic prioritization of limited resources; anticipating impacts of climate change; integrating efforts across phases and zones; ensuring health equity; and addressing evidence gaps through targeted research. Table 1.

**Table 1:** Critical themes for healthcare resilience to tropical cyclones.

Theme	Key challenges	Examples	Strategies
1. Continuity of care for vulnerable populations	Disruptions to dialysis, oncology, chronic medications post-cyclone.	- Post-Maria dialysis shortages (Kishore 2018) - Radiotherapy delays (Nogueira 2019)	Pre-storm medication stockpiles; alternate care sites for chronic conditions.
2. Proactive resilience versus reactive response	Overreliance on post-disaster resources; climate change intensifies risks.	- 41% rise in hospital damage (Huang 2023) - Supply chain failures (Hurricane Maria IV shortages)	Climate-hardened infrastructure; regional resource-sharing agreements.
3. Resource constraints & prioritization	Competing demands during multi-hazard events (cyclones + wildfires/heatwaves).	- Staff burnout post-Harvey - ED overcrowding limits surge capacity	Cross-credentialing staff; tiered crisis standards of care.
4. Climate change intensification	Rising storm intensity, sea levels, and poleward shifts (Kossin 2014).	- 71% of high-risk hospitals in LMICs - Flooded coastal facilities (Tarabochia-Gast 2022)	Build hospitals above 500-year flood levels; use local climate projections for siting.
5. Cross-phase & cross-zone integration	Fragmented coordination between regions/phases (Before-During-After).	- Evacuation chaos during Sandy - Telemedicine gaps (Rubin 2014)	Unified command systems (HEICS); pre-negotiated patient transfer protocols.
6. Health equity in interventions	Marginalized groups face higher mortality (e.g., elderly, disabled, low-income).	- Post-Katrina racial disparities - Indigenous knowledge exclusion (Choudhury 2021)	Culturally tailored outreach; prioritize vulnerable groups in evacuation plans.
7. Evidence gaps & research needs	Limited data on long-term morbidity and LMIC impacts (Dresser 2022).	- Excess deaths in Puerto Rico (Kishore 2018) - Mental health PTSD studies (Kim 2021)	Fund longitudinal studies; expand LMIC research partnerships.

Intravenous (IV), Emergency Department (ED), Low-Middle Income Countries (LMIC), Post-Traumatic Stress Disorder (PTSD).

## Background

The risk profile of tropical cyclones, the vulnerability of populations in harm’s way, and societal response capacity relative to these needs are trending in concerning directions.^5^ First, climate change likely means increasingly intense tropical cyclones with higher rainfall potential, higher risk of rapid intensification leading to reduced warning periods, and reduced translocation speeds, which can lead to longer periods of direct impact and thus greater destruction and disruption.^12^^,^^13^ In addition, in some ocean basins the locus of maximum intensity appears to be moving poleward,^14^ meaning that healthcare systems with little historical experience preparing for or responding to tropical cyclones may face increasing hazard exposure in future years. Sea level rise resulting from climate change will lead to increasing coastal flooding and storm surge risks, particularly as sea level rise may exceed 1.3 m later in the 21st century, with substantial implications for healthcare facilities in coastal areas.^15^

Second, healthcare systems in regions that have undergone demographic transition can expect to care for aging, medically complex patients who need continuity of care and access to care in the aftermath of future tropical cyclones. While direct injuries from flooding and wind damage will continue to result in healthcare utilization and must be prepared for,^16^ maintaining continuity of care for medically fragile patients who rely on outpatient hemodialysis services, oncology infusion services, wound care services, availability of oxygen, and availability of insulin and a wide variety of other critical medications can present a much larger challenge if large populations of medically complex patients are left without housing or access to electricity.^17^^,^^18^ Tropical cyclone disasters are linked to reduced survival among cancer patients undergoing radiotherapy (hazard ratio 1.19 for death among patients exposed to hurricane disasters)^4^ and patients on hemodialysis (hazard ratio 1.13).^3^ Failure to maintain connectivity between patients and essential healthcare services on which they depend is a plausible factor in the prolonged increases in morbidity and mortality that have been found to follow tropical cyclone events in recent research studies.^8^^,^^18^

Third, societal response capacities have limits. Response-based approaches to addressing healthcare impacts from tropical cyclones assume the availability of response resources, but with other climate-responsive hazards including floods, wildfires, heat waves, and extreme weather escalating in frequency and intensity, response resources may be stretched thin and operational tempos may become unsustainable.^12^

**Fig. 2:**
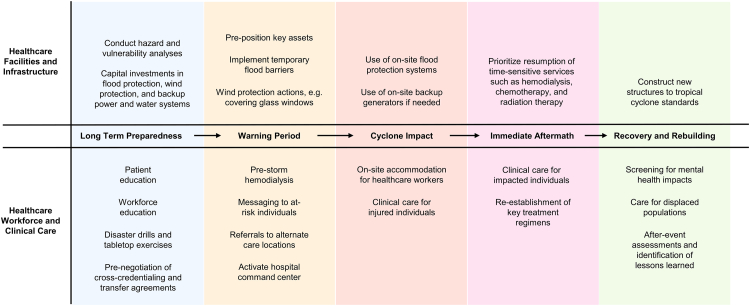
Adaptive actions before, during, and after tropical cyclone impact.

While understanding the scope and nature of cyclone threats establishes the challenge, healthcare systems must first ensure their physical foundations can withstand these increasingly severe events. Infrastructure resilience forms the essential first layer of defense, as without functioning facilities, power, water, and supply chains, even the most well-prepared clinical teams cannot deliver effective care to vulnerable populations. Below we outline adaptive actions before, during and after tropical cyclone impact. Fig. 2.

## Healthcare facilities and infrastructure resilience

Tropical cyclones commonly threaten healthcare facilities infrastructure, and operations, as well as patients' ability to access healthcare services. In the immediate aftermath of tropical cyclone events, healthcare systems are seeing more severe damage to their physical infrastructure from flooding, wind, debris, and loss of utility services (electricity and water), as well as loss of healthcare access for patients, staff, and vendors due to transit and roadway disruptions, destruction of medical storage and supplies, injuries and illness among healthcare personnel, supply chain interruptions and other effects. These cyclone impacts can be prolonged and are leading to larger and longer effects on community recovery, including decreases in health services for the community at large, food and water scarcity, decreased availability of adequate housing, population displacement, conflict, environmental degradation, increases in vector-borne diseases, worsening exacerbations of chronic diseases, and decreased mental health and well-being.

The magnitude of threats to hospital infrastructure is increasing. A 2023 report documents that damage to hospitals from severe weather has increased globally by 41% between 1990 and 2020. By the end of the century, 1 in 12 hospitals worldwide may be at high risk of partial or total shutdown from extreme weather events, with 71% of the hospitals at highest risk in low- and middle-income countries.^19^

Accurately assessing risk is the essential first step towards addressing infrastructure risks from cyclones and developing effective response and recovery plans. Communities and the health centers that serve them need access to scientifically accurate data that reflects the most up to date predictions about the anticipated effects of climate change in their location, including precise local flood maps and detailed predictions about the wind speeds and temperature ranges that they may experience in future years. These data are necessary to determine emergency supply and transportation routes and to identify and prioritize local climate resilience efforts.^11^

Assessment of cyclone risks and consequences must consider both short-term and long-term planning hazard vulnerability analyses. In the short-term, risk analyses inform risk reduction activities, such as purchasing flood-barriers, hardening utility connections, and other actions that will decrease the near-term likelihood of severe damage or loss of function of the healthcare facility.^11^ In the long-term, risk projections must inform the location, design, and construction of new healthcare infrastructure as it is built. The lifespan of a hospital building may exceed 50 years; to avoid construction that becomes vulnerable by design, new healthcare facilities must be built to withstand the anticipated effects of cyclones decades in the future.

Unfortunately, access to accurate data for healthcare risk assessment remains a major challenge. While increases in tropical cyclone intensity and rainfall have been well described,^20^ existing climate assessments commonly lack the local-level details of potential flood elevations or maximum windspeed estimates needed to inform facility-specific planning.^21^ Historical cyclone severity data may underestimate the magnitude of future tropical cyclone hazards; current building codes, which are typically informed by historical data, may be insufficient.^22^ Healthcare emergency planners must work with community and academic leaders, climate scientists, and other experts and leverage data sourced from governments, utilities, insurance providers, and environmental agencies to identify scientific predictions of precipitation, flooding, and windspeed severity, as well as to identify predictable vulnerabilities among utility service, transportation, and other key partners. Examining probabilistic data such as 100-year and 500-year storm maps may be useful for determining institutional risk tolerance, but using consequence-based maps, which can show worst-case scenarios under certain conditions, are often more useful when planning to protect “never-fail” services, such as emergency, surgical, critical care, childbirth and other critical healthcare services.^23^

Supply chains deserve special consideration. Facilities that are directly impacted by tropical cyclones or that depend on vulnerable transport infrastructure may be unable to receive shipments of medications, medical supplies, oxygen, fuel for backup generators, food, and other critical items for prolonged periods; this has led to reduced functioning or closure of healthcare facilities ranging from major hospital to community pharmacies following major storms.^24^ Facilities thousands of miles from tropical cyclones have experienced supply chain disruptions, including shortages of intravenous fluids following Hurricane Maria and Hurricane Helene.^11^^,^^25^^,^^26^

As healthcare systems vary in size and complexity, it is difficult to quantify the costs of actions such as pre-storm medication stockpiling and the expected impact of these actions on mortality reduction. As a result, there is a paucity of cost-benefit analysis data available regarding storm impact on hospitals. One survey of hospital leadership supports the idea that the cost of training and disaster supplies impacts hospitals positively.^27^ Due to the severity and global impact of the recent COVID-19 pandemic, more work has been done to highlight the cost effectiveness of stockpiling pandemic related drugs and ventilators.^28^ From these data, it can be inferred that similar preparedness for other devastating disasters may also be effective. Moreover, one group of researchers has proposed a game-theory model for hospitals to meet the surge in demand for medical supplies that a disaster creates at the lowest cost possible.^29^

Although robust infrastructure creates the physical environment for healthcare delivery, it is the clinical staff, protocols, and adaptive actions to maintain access to care that ultimately support positive patient outcomes during cyclones.

### Healthcare workforce and clinical care

Healthcare systems must develop clinical and operational preparedness strategies that leverage investments in structural readiness while accounting for their potential limitations during disasters. Without trained personnel and flexible protocols, even the most resilient facilities cannot maintain continuity of care for vulnerable populations.^30^

Prior to a storm, healthcare systems have numerous opportunities to reduce risk to their patients through proactive action. Scheduling patients for dialysis sessions immediately before tropical cyclone landfall increases the time available to reestablish outpatient dialysis after a storm, and has been shown to be protective, with reduced odds of Emergency Department (ED) utilization, hospitalization, and death in the 30 days following a storm.^31^ Outpatient clinics can provide education on individual risk reduction strategies, including readiness for evacuation and health maintenance in post cyclone environments.^32^ Pre-arranging for continuity of critical health services such as cancer treatments, dialysis, and other time sensitive care at alternate sites also has substantial potential to reduce health harms from these storms.^3^^,^^4^

Staffing strategies must also be considered. While staffing needs before, during, and after a disaster may increase, staff availability may decrease. Healthcare workers may have difficulty driving to work due to road closures, debris, flooding, or other safety concerns; hospitals can work with staff to arrange on-site housing for the duration of the disaster. Employees may be responsible for family members, pets, or have other obligations that prevent them from coming to work^33^^,^^34^; again, hospitals can step in by helping connect these family members with resources and a safe place to stay during the storm, which might include on-site housing. When a hospital prepares for a disaster in this way, it must broaden its food, and water supplies to be inclusive of these additional people. If physical space is limited, staff-related housing must be balanced against capacity to handle an influx of patients.^35^

Healthcare systems that are not in the impact zone and do not experience direct effects from wind and water can nonetheless experience substantial impacts related to tropical cyclones affecting nearby or distant locations. Large-scale evacuations from hurricane impact areas can lead to transient increases in healthcare utilization elsewhere, a contingency for which health systems in adjacent regions should be prepared. Activation of personnel for response activities can also lead to reduced staffing levels at otherwise unaffected sites.^11^^,^^35^

Both during and after a storm, hospitals should prepare for a surge of patients, including both acutely ill patients as well as those simply seeking shelter from the storm or its aftermath. In the week following Hurricane Sandy in New York, for example, there was an increase in patients, particularly older adults, coming to the ED for homelessness or housing insecurity.^36^ Tropical cyclones impact large swathes of the surrounding environment and infrastructure. While for localized mass casualty incidents a hospital may be able to create room inside the hospital by discharging patients, in a tropical cyclone disaster, expedited discharges will often be unsafe. Hospitals must be ready to convert unused space into clinical or shelter areas for patients and those seeking shelter or power for medical devices who may become patients themselves if their health maintenance needs are not met.

While the acute phase of a storm may see a surge in trauma and critical care, chronic disease management and mental health support will likely play a vital role in the subacute and recovery phase, if not earlier. Direct trauma from the storm may cause a surge in head injuries, fractures, and other blunt traumas,^10^ necessitating that hospitals be prepared for increased operating room utilization with appropriate operative staffing and critical care personnel to support patients post-operatively. However, many more patients may come to the facility seeking medication refills, dialysis, wound care, or management of chronic medical problems for which they no longer have access to their standard outpatient care. Helping patients care for their chronic medical problems will require additional medical staff and supplies but will be crucial in keeping these patients stable. Hospitals, and in particular EDs, play a key role in public health during disasters as they are the point of healthcare access and safety nets for all people, especially vulnerable populations.

Tropical cyclones are associated with an increased mental health burden among patients, especially post-traumatic stress disorder; early mental health support should be included in a hospital's preparedness plan.^10^^,^^16^ Hospitals should use tools developed specifically for psychological first aid for people who have experienced disasters, such as the Psychological First Aid Manual from the National Center for Post-Traumatic Stress Disorder (PTSD).^37^^,^^38^

There may come a time during a tropical cyclone when the hospital is forced to evacuate all patients and personnel. This may be due to storm severity, flooding, or failing support infrastructure such as electricity generators or dwindling medical supplies. To prepare for this contingency, hospitals must have protocols in place for safe evacuation and staff must be trained in these protocols.^39^^,^^40^ Clear communication to staff and patients is necessary, and a command structure, such as the Hospital Emergency Incident Command System (HEICS), should be in place and should be activated in times of crisis to provide order to the entire disaster response, especially evacuation.^41^, ^42^, ^43^, ^44^ Most importantly, the hospital needs clear communication and coordination with outside agencies to guide where patients will be evacuated to. For example, if a hospital is part of the US National Disaster Medical System, federal coordinating centers will help with patient redistribution.^41^^,^^42^

Telemedicine may have utility when providers cannot reach a cyclone-affected hospital. It is already being used with success by hospitals with limited resources for stroke,^45^ psychiatry,^46^ and critical care consultations.^47^ When used correctly, telemedicine can be diagnostically reliable and maximally utilize limited resources.^45^, ^46^, ^47^, ^48^ The COVID-19 pandemic saw rapid expansion of telemedicine, with benefits in patient monitoring, communication with patients, and a decrease in unnecessary exposure of healthcare workers to COVID.^49^ Telemedicine can also be used to help cognitively off-load physicians in the hospital by providing guidance for patient care remotely.^49^ In disaster scenarios such as a tropical cyclone, hospitals may find themselves inundated with patients, and even if healthcare workers can't physically reach the hospital, telemedicine would allow them to provide aid remotely. Health systems impacted by a TC will need to plan for internet connectivity challenges and consider the use of satellite systems to support telemedicine operations.^50^

## Governance and leadership

Effective healthcare system response to tropical cyclones requires robust governance structures and leadership frameworks that can adapt to rapidly evolving crisis conditions. While preparedness involves resources and supplies, resilience during disasters demands clear command hierarchies, regulatory flexibility, strategic partnerships, sustainable funding, and leaders equipped with crisis management competencies.

### Command and control

Command and control systems form the backbone of healthcare disaster response, with the (HEICS) serving as the predominant framework in many settings. This standardized approach enables healthcare facilities to align their incident management structure with broader emergency response frameworks.^41^^,^^51^ During tropical cyclones, a unified command structure facilitates rapid decision-making and resource allocation while maintaining clear lines of authority. Research demonstrates that healthcare systems implementing HEICS experience fewer communication breakdowns and more efficient resource utilization during prolonged disaster responses compared to ad hoc command structures.^51^^,^^52^

Implementation challenges remain, however, particularly in regions with fragmented healthcare systems or limited disaster response experience. Hospitals must designate personnel for key command positions before disasters strike and establish redundancy in leadership roles to accommodate prolonged operational periods, which is particularly important during extended tropical cyclone events that may involve evacuation, sheltering, and recovery operations spanning weeks.^52^

### Regulatory frameworks

Regulatory environments critically influence healthcare system cyclone resilience. Rigid regulations impede effective disaster response; thus, jurisdictions have established emergency waivers enabling temporary flexibility in capacity limits, staffing requirements, alternate care site establishment, and crisis standards implementation. Pre-emptive regulatory adjustments are essential for cyclone response given storm predictability.^53^ Regulatory frameworks should mandate facility hardening requirements in vulnerable regions, including elevation standards, structural specifications, and redundant utilities. The Pan American Health Organization (PAHO) Smart Hospitals program exemplifies comprehensive regulatory integration of disaster resilience into healthcare infrastructure standards, demonstrating proactive resilience enhancement through anticipatory policy frameworks.^54^

### Public-private partnerships

Public-private partnerships constitute essential healthcare resilience mechanisms, enabling resource sharing, expertise exchange, and risk distribution. During cyclones, these collaborations facilitate multi-facility patient evacuations, integrated supply chain management, and healthcare personnel transportation. Effective implementations include pre-established transportation agreements, hotel partnerships for patient relocation, and pharmaceutical distribution arrangements.^55^ Success determinants encompass clearly delineated responsibilities, formalized pre-disaster agreements, regular drills, and communication protocols.

### Funding mechanisms

Healthcare systems face persistent funding challenges for cyclone preparedness, where economic tensions exist between immediate operational demands and long-term resilience investments.^56^ Effective financial approaches utilize multiple mechanisms including preparedness grants, insurance instruments, capital improvement allocations, and contingency funds.^57^ Emerging innovative financing includes healthcare-specific catastrophe bonds, resilience credits incentivizing preparedness, and public-private partnerships.^57^^,^^58^ Climate adaptation funding increasingly prioritizes healthcare resilience, acknowledging its critical community recovery role.^56^ Financial strategies must of course balance immediate response liquidity with sustained structural and operational resilience investments. Therefore, it is extremely important to try to estimate the potential costs associated with being under-prepared, so as to justify investments for resilience. Guenther and Balbus’ Sustainable and Resilient Healthcare Facilities Toolkit offers a general framework to identify the types of costs of being ill-prepared and lists the “bottom line” damages for healthcare when it is unable to prevent or mitigate damage.^59^ The 2022 report of the European Environment Agency presents a detailed listing of the various monetary valuation methods and metrics that may be used when trying to quantify the costs of adaptation versus the costs of inaction.^60^

### Decision-making protocols

Tropical cyclones create complex decision-making scenarios for healthcare leaders, including whether to shelter in place or evacuate, how to allocate limited resources, and when to implement crisis standards of care.^61^, ^62^, ^63^ Effective decision-making protocols incorporate clear triggers based on objective criteria rather than subjective assessments. These protocols should define specific thresholds for activation of emergency operations, evacuation initiation, and implementation of altered care approaches.

Decision support tools that integrate real-time data on storm trajectory, facility status, patient acuity, transportation availability, and receiving facility capacity can enhance decision quality during high-stress periods.

### Crisis leadership competencies and training

Effective cyclone response management requires healthcare leaders to develop core competencies: situational awareness, decision-making under uncertainty, crisis communication, resource prioritization, and emotional resilience, balancing immediate tactics with strategic recovery planning. Leadership preparation necessitates both technical emergency management knowledge and practical simulation experience.^64^ System-wide disaster exercises incorporating realistic cyclone scenarios identify leadership deficiencies and organizational vulnerabilities preemptively. This training must encompass all decision-making levels from executives to operational leaders.^65^^,^^66^

Regional training collaboratives foster inter-organizational relationships while promoting standardized approaches across systems. As climate change intensifies cyclone threats, crisis leadership development represents a high-yield resilience investment.^66^ While governance structures facilitate organizational adaptation, comprehensive healthcare resilience transcends institutional boundaries, requiring integration with community resources, perspectives, and needs. This community integration transforms isolated institutional readiness into cohesive regional resilience systems supporting vulnerable populations throughout the cyclone continuum.^66^

## Community integration and social resilience

Community engagement serves as a foundation for fostering resilience by ensuring that strategies align with community priorities and capacities. Effective engagement includes conducting regular community meetings, leveraging local leadership structures, and disseminating information through culturally relevant materials. These strategies enhance community participation and promote power sharing, equity, and flexibility in disaster preparedness and response. Community-based participatory research (CBPR) represents an equitable research methodology that establishes partnerships between academic researchers and community stakeholders across all phases of investigation, including problem identification, study design, data collection, and result dissemination. This model centers community priorities and local knowledge systems while seeking to reduce health inequities and enhance population health outcomes through collaborative decision-making processes and community-driven interventions.

This approach builds scientific and community capacity, ensuring the use of research findings for practical applications. Community-based disaster drills also play a crucial role in fostering preparedness, with initiatives such as housing family pets alongside staff families in hospitals during emergencies to promote bonding and resilience.^2^

Vulnerable populations are at greater risk for poor physical and psychological health outcomes during and after disasters as they are less likely to take self-protective actions.^67^ Any group of individuals that have a heightened threat level due to their condition are considered a vulnerable population, this includes but is not limited to: pregnant women, Neonatal Intensive Care (NICU) babies, newborns and mothers, people with disabilities, elderly individuals, and patients with chronic illnesses. Pregnant women, NICU babies, and their mothers have been prioritized for evacuation in past crises.^2^ People with disabilities and elderly individuals face heightened risks during disasters due to the intense displacement procedures that can alter their state.^1^ Chronic illness patients, such as those undergoing radiotherapy for lung cancer, are also at risk due to disrupted electrical power. Treatment delays are one of the preventable hurricane-related disruptions, underscoring the need for targeted interventions.^4^ Addressing treatment delays and ensuring continuity of care can mitigate such risks.

Communication protocols are a fundamental component of disaster resilience. Before a disaster, establishing reliable communication systems ensures timely dissemination of warnings and preparedness measures.^68^ Public trust in information sources and the delivery medium is critical. During crises, updates regarding shelter availability and emergency contacts facilitate coordinated responses. In the aftermath, recovery resources, disease prevention, and mental health support must be readily accessible. Deploying mental-health nurses and counseling psychologists is crucial in mitigating psychological distress.^69^

Social support networks significantly enhance resilience. Social media platforms serve as vital tools in disaster response, as evidenced during Hurricane Katrina.^70^ Nonprofit organizations play a pivotal role in linking communities with external resources, including governmental agencies and diaspora networks. Inter-neighborhood collaboration, as observed post-Katrina, accelerates recovery compared to isolated communities.^71^ Social capital fosters recovery through shared values, expanded networks, and strengthened community partnerships.^70^ Moreover, social support has been shown to mitigate the negative psychological effects of disaster-related trauma.

## Health equity in disaster: addressing disparities in cyclone impacts and recovery

Health equity implementation requires targeted outreach to marginalized populations, culturally appropriate communications, and equitable resource distribution. Institutional frameworks frequently exclude indigenous knowledge systems from resilience planning, imposing exogenous resilience conceptualizations.^71^ Disaster impacts disproportionately affect historically underserved communities, necessitating interventions addressing healthcare barriers and infrastructure investments. Public health institutions require structural transformation to align with community-partnered approaches.^72^ While essential, community integration initiatives must synchronize with broader health system coordination mechanisms connecting multiple facilities and jurisdictions to establish regional resilience supporting communities when local capacities are exceeded, maximizing resource utilization across geographic and temporal boundaries.

## Health system coordination- cross-phase, cross-zone integration: coordinating resilience efforts across time and space

There is a distinct difference between healthcare system preparedness and readiness. While hospitals may have adequate supplies, medical personnel, and inpatient beds to care for victims of a disaster, commonly referred to as the “space, staff, and stuff” of preparedness, do they possess the final “S” that reflects level of readiness: organizational structure? This refers to the integration of pre-event policies and clinical care capabilities necessary for effective and efficient disaster response.

While preparedness involves strategies around resources, personnel, and supply chain management, readiness is dependent on the proactive steps taken as an integrated healthcare system to ensure hospitals can make the transition from daily operations to crisis response as quickly and efficiently as possible. In natural disasters such as cyclones, particularly in resource-limited countries, steps taken to enhance healthcare system readiness lead to a quicker and less resource-dependent response.^73^

Improving readiness includes taking actions to coordinate regional healthcare system frameworks that maximize individual hospital resources and build local capacity. Disasters begin at the local level and the scene of the event. Depending on the size of the disaster and its impact on the community, the effects ripple out like waves from a stone dropped in water, the heavier the stone the further the waves travel. This is the case for both impact and response needs, and therefore the old axiom “all disasters are local” holds true.^74^ As the impact of the disaster increases more outside resources are required, however, they also require more time to arrive. Therefore, the readiness steps proactively taken are most important for the acute-phase of healthcare system disaster response.

One hurdle in effective readiness is the current global ED and hospital overcrowding crisis.^75^ When EDs are operating at levels far beyond their capacity on a daily basis, surge response capability is greatly hindered when disaster strikes. Response steps such as reverse triage, the proactive emptying of emergency department beds when a disaster strikes, are difficult to implement because there are no open beds for patients to be admitted to. Under these circumstances, healthcare systems must proactively develop innovative solutions such as resource sharing agreements and alternate care sites prior to disaster events.^76^

Perhaps the most important action to take for enhanced healthcare system readiness is to exercise crisis response frequently, including community-wide drills. The more tabletop and full-scale exercises hospitals can conduct, including coordinated drills with other community healthcare assets, the more efficiently the system will operate in times of disaster.^11^^,^^16^

Pre-negotiating transfer agreements and protocols, either within or across systems, is essential for effective coordination. Similarly, cross-credentialing providers and nurses so that they can follow their patients if they have to evacuate their own hospital creates continuity in care during evacuations and ensures patients retain access to providers familiar with their medical history and needs.

While immediate coordination during cyclone response saves lives, the transition to long-term recovery represents a critical yet often neglected phase that determines ultimate health outcomes. The following section addresses the unique challenges of the recovery phase and strategies to reduce long-term health impacts.

## Recovery and long-term health impacts

Evidence indicates significant health impacts occur during recovery periods rather than during tropical cyclones, and that long-term impacts on mortality may extend for up to 15 years after major storms.^77^ This epidemiological shift reflects both improved early warning systems reducing immediate trauma deaths and the vulnerability of medically complex populations to care disruptions.^8^^,^^78^ Healthcare systems require comprehensive recovery strategies addressing four domains: infrastructure restoration, care access reestablishment, workforce recovery, and integration with community-wide efforts addressing social determinants of health.

## Inside the impact zone

Inside the impact zone, healthcare facilities must transition from emergency operations to staged recovery while continuing to meet elevated healthcare needs. This often requires managing a dual burden: addressing acute disaster-related conditions while simultaneously restoring capacity for routine and chronic care management.^5^ Healthcare facilities that remain operational during a storm often experience extended periods of higher-than-normal patient volumes, as nearby damaged facilities remain closed. This sustained surge requires careful resource management, staff rotation to prevent burnout, and phased restoration of elective services.^78^^,^^79^

For facilities that experienced damage or evacuation, reestablishing operations requires systematic assessment and remediation. This includes not only physical repairs to infrastructure but also careful environmental monitoring to address potential contamination, mold growth, or other health hazards that can develop in storm-damaged buildings.^80^ The reopening of damaged facilities should follow a prioritized approach that considers both community needs and operational feasibility, with emergency services often reopening first, followed by inpatient capacity, and finally outpatient and elective services.^80^ Time-sensitive outpatient services, such as hemodialysis, chemotherapy, and radiation oncology services, should also be prioritized.

Healthcare workforce recovery deserves special attention, as personnel often experience dual impacts as both disaster responders and disaster victims. This combination creates high risk for burnout, compassion fatigue, and post-traumatic stress.^81^ Healthcare systems must develop comprehensive staff support programs that address both professional and personal recovery needs. These may include housing assistance, childcare support, mental health services, flexible scheduling during recovery periods, and financial assistance for personal recovery costs.

A critical recovery challenge involves reconnecting displaced patients with their healthcare providers and maintenance medications. Research following Hurricane Maria in Puerto Rico demonstrated that medication interruptions contributed significantly to excess mortality, particularly among patients with chronic conditions such as hypertension, diabetes, and heart failure.^8^^,^^82^ Healthcare systems must develop systematic approaches to locate displaced patients, assess their medication needs, and provide bridge prescriptions until regular care can be reestablished. Mobile health units, temporary clinics in shelters, and partnerships with pharmacies in receiving areas can help address these needs.^8^^,^^83^

## Outside the impact zone

Healthcare systems in receiving areas must prepare for both immediate evacuee influx and extended displacement needs through medical record access systems, coordination with disaster case management for vulnerable populations, and contingency planning for prolonged capacity demands. Post-cyclone mental health sequelae—including elevated rates of anxiety, depression, PTSD, and substance use disorders—necessitate integrated screening, primary care support, and sustained resource allocation beyond physical infrastructure.^84^

Healthcare actions must synchronize with community-wide efforts addressing social determinants of health, including housing, transportation, utilities, food security, and economic revitalization. Healthcare should engage in comprehensive planning that prioritizes accommodations for vulnerable populations, healthcare access routes, and disparity reduction initiatives.^8^

Epidemiological studies document persistent post-cyclone health disparities affecting socioeconomically disadvantaged populations.^8^^,^^85^ Recovery planning must incorporate equity-focused interventions including equitable resource allocation, targeted outreach to displaced marginalized communities and individuals, and elimination of administrative barriers to care reestablishment.^86^ These recovery considerations, aligned with broader infrastructure, clinical, governance, and coordination frameworks, inform prioritized interventions addressing critical vulnerabilities within existing resource constraints (Box 1).

Box 1 **Recommendations and future directions.**

We propose the following priority recommendations organized by implementation timeframe and resource requirements:

**Immediate actions (low resource)**

1. Develop and regularly exercise healthcare evacuation and surge capacity plans that address the specific needs of medically vulnerable populations.
2. Establish cross-institutional agreements for patient transfers, resource sharing, and staff cross-credentialing before disasters occur.
3. Integrate continuity of care planning for chronic conditions into disaster preparedness efforts, particularly for dialysis, oxygen-dependent, and medication-dependent patients.
4. Implement community engagement strategies that incorporate local knowledge and prioritize equitable participation in resilience planning.
5. Provide patient education and counseling on individual risk reduction strategies, particularly for medically or socially vulnerable patients.

**Medium-term priorities (moderate resource)**

1. Create regional healthcare coordination centers that integrate emergency management, healthcare facilities, and community organizations across jurisdictional boundaries.
2. Develop redundant supply chain systems for critical medical supplies, medications, and equipment with particular attention to items known to experience shortages following disasters.
3. Implement healthcare facility hardening measures based on updated climate projections rather than historical data, following PAHO Smart Hospitals guidelines where appropriate.
4. Establish healthcare workforce resilience programs that address both professional and personal impacts of disasters.

**Long-term investments (high resource)**

1. Relocate critical healthcare infrastructure out of high-risk coastal or riverine flood zones, or implement comprehensive flood mitigation measures where relocation is not feasible.
2. Develop integrated health information systems that maintain accessibility during disasters and facilitate continuity of care for displaced populations.
3. Build distributed healthcare delivery networks that maintain functionality even when specific nodes are compromised.
4. Establish dedicated funding mechanisms for healthcare resilience that balance immediate needs with long-term adaptation to climate change.

## Building the evidence base: research priorities for healthcare resilience

While the highest mortality burden from tropical cyclones will likely continue to be in low- and middle-income settings,^87^ research on the mortality impacts of tropical cyclones has been disproportionately conducted in higher income settings.^88^ From 1985 to 2019, less than 1% of global deaths attributed to TCs occurred in the United States, yet more than half of all articles on TC mortality have focused on US contexts.^88^ Broadening the research base to include a greater number of studies in low- and middle-income settings in these regions is an urgent priority. Mechanisms to achieve this include professional development programs for early and mid-career researchers,^89^ targeted grant funding,^90^ and investment in research infrastructure.^91^

It is also important to continue to improve understanding of the health impacts of tropical cyclones, particularly with respect to their long-term effects^77^ and their effects in patient populations with different baseline characteristics, including varying levels of medical complexity, vulnerability and medical device dependence.^8^ Studies of anticipated future risks and healthcare needs in the context of climate change, aging populations, and the double burden of disease are important to guide long-term policy.

## Resilience metrics

Development and validation of healthcare resilience metrics specific to tropical cyclones can help track progress and guide resource allocation. Existing resources, including the World Health Organization (WHO) Climate Change and Health program’s Resources for Measuring the Climate Resilience of Health Systems^92^ and the list of resilience actions related to on the impacts of hurricanes of healthcare facilities in the US Office of Climate Change and Health Equity’s Climate Resilience for Health Care Toolkit,^93^ provide a starting point. Future work will need to focus on establishing specific metrics and targets that are sufficiently granular to measure numerically within individual institutions. These could include, for example emergency services, surgical capacity, mental health services, facility offline time, duration of interruptions in care.

Tropical cyclone resilience assessments, actions, and metrics related to dialysis provide an illustrative example of the challenges inherent in this process, which involves considering granular details of healthcare in the context of a strategic-level threat. In one study, patients with end stage renal disease who withdrew from hemodialysis for psychosocial reasons had a median survival of 10 days without dialysis,^94^ with 40% experiencing death within a week; other studies have shown survival times of 3–10 days after discontinuation of dialysis.^95^^,^^96^ Dialysis services are often interrupted by tropical cyclones; providing dialysis just before hurricane landfall has been shown to reduce the odds of ED utilization, hospitalization, and death. Metrics such as rates of pre-storm dialysis, duration of outpatient dialysis disruption, and post-storm ED presentations for dialysis needs could be applied to help administrators track and improve performance of a healthcare system with respect to this issue, but are not routinely tracked in many systems. The WHO Resources described above do not mention “dialysis”, but do describe the need to assess “weaknesses in current technical and health service capacities and necessary adaptations”.^92^ The OCCHE Toolkit recommends that health systems “consider preemptive planning for patients who use electricity-dependent Durable Medical Equipment (DME)” and “develop a continuity of care plan listing essential clinical services that will be provided at different tiers of utility disruption,” and suggests the use of pre-storm dialysis and mobile health units to provide access to post-disaster dialysis services, but does not identify specific metrics to assess the accessibility, utility, or cost-effectiveness of such programs.^93^ Future work focused on the implementation and assessment of resilience actions will be necessary in order to identify and validate metrics suitable for widespread adoption within healthcare systems facing tropical cyclone risks.

Resource Allocation Decision Making Frameworks and Low and Middle Income Countries (LMIC) Research Methodologies.

Resource allocation decisions in tropical cyclone preparedness require systematic frameworks that account for opportunity costs and competing priorities, particularly in resource-constrained LMIC settings. Few studies have examined this empirically.^97^, ^98^, ^99^ However, LMIC research reveals additional methodological considerations essential for effective implementation. Studies of the Typhoon Haiyan and supertyphoon Odette in the Philippines exemplifies culturally appropriate approaches, utilizing semi-structured interviews with local health staff that incorporated Filipino cultural values to identify accessibility, safety, and emotional impact domains.^100^^,^^101^ Future LMIC tropical cyclone research should integrate evidence based frameworks with mixed-methods approaches that combine quantitative surveys with qualitative interviews, prioritize community partnerships established before disasters occur, and develop culturally adapted instruments through systematic validation processes.

There remains a need to develop standardized costing methodologies for cross-study comparison and conduct longitudinal studies measuring preparedness investment impacts.^102^ Clarke et al. (2022) found that costing methods varied substantially among LMIC studies from US 1.6 Billion per year to improve disaster preparedness capacity to US$ 43 billion per year, making comparisons of absolute costs or per-capita costs difficult.^103^ More empirical studies are needed to validate tiered intervention outcomes across different healthcare settings.

## Conclusion

Tropical cyclones present increasing threats to healthcare systems amid climate intensification and demographic vulnerability shifts. This analysis yields two critical findings: declining immediate mortality contrasts with increasing long-term morbidity from disrupted healthcare access, and effective resilience requires multidimensional coordination across infrastructure, clinical, governance, and community domains. Implementation challenges include resource constraints, overcrowding limiting surge capacity, system fragmentation, regulatory inflexibility, and insufficient climate data. Research priorities include addressing gaps in low/middle-income settings, continuity-of-care interventions, resilience metrics, and future healthcare needs projections. Healthcare institutions must transition from reactive response to proactive resilience strategies to safeguard vulnerable populations in an intensifying climate crisis.

## Contributors

AJH, led the project, designed the project, provided oversight, reviewed the literature, wrote the first draft, coordinated edits, provided final approval of the version to be published, and was responsible for the decision to submit the manuscript. CD, SG, APH, PB, and GC, reviewed the literature, contributed to the drafting of the work, critically reviewing it for intellectual content and provided approval of the final version to be published.

## Declaration of interests

Dr Dresser reports gifts to Harvard TH Chan School of Public Health from Biogen and Johnson & Johnson to support development of climate resilience educational materials which include information about hurricane preparedness. We declare no other competing interests.

Dr. Biddinger reports that he is a board member of the American Red Cross of Massachusetts an unpaid role.
